# (*E*)-*N*′-(2-Chloro-5-nitro­benzyl­idene)-3-meth­oxy­benzohydrazide monohydrate

**DOI:** 10.1107/S1600536811020769

**Published:** 2011-06-11

**Authors:** Shi-Yong Liu, Xiao-Ling Wang

**Affiliations:** a College of Chemistry and Pharmacy, Taizhou University, Taizhou Zhejiang 317000, People’s Republic of China; b Department of Chemistry, Liaoning Normal University, Dalian 116029, People’s Republic of China

## Abstract

In the hydrazone mol­ecule of the title compound, C_15_H_12_ClN_3_O_4_·H_2_O, the two benzene rings form a dihedral angle of 3.6 (1)°. In the crystal structure, the solvent water mol­ecules are involved in the formation of inter­molecular N—H⋯O and O—H⋯N hydrogen bonds, which link the mol­ecules into double ribbons extending along the *b* axis. Inter­molecular π–π inter­actions between the aromatic rings [centroid–centroid distances = 3.712 (3) and 3.672 (3) Å] link these ribbons further into layers parallel to the *ab* plane.

## Related literature

For the crystal structures of hydrazones recently reported by us, see: Liu & You (2010*a* ▶,*b* ▶,*c* ▶); Liu & Wang (2010*a* ▶,*b* ▶); Sun *et al.* (2011 ▶).
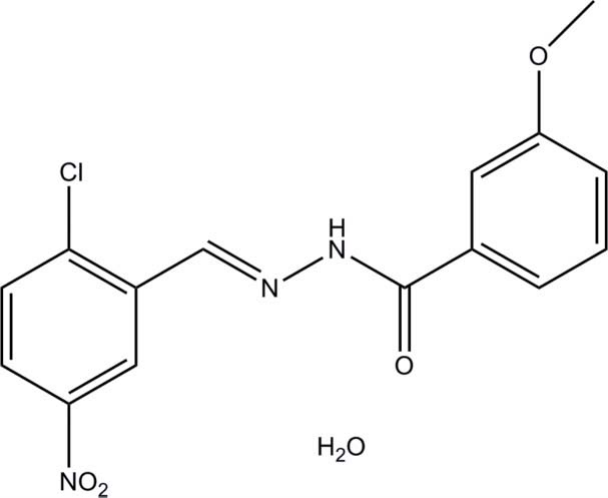


## Experimental

### 

#### Crystal data

C_15_H_12_ClN_3_O_4_·H_2_O
*M* *_r_* = 351.74
Triclinic, 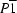

*a* = 7.176 (2) Å
*b* = 7.179 (2) Å
*c* = 15.395 (4) Å
α = 83.820 (18)°
β = 89.953 (18)°
γ = 80.190 (18)°
*V* = 776.8 (4) Å^3^
*Z* = 2
Mo *K*α radiation
μ = 0.28 mm^−1^
*T* = 298 K
0.23 × 0.21 × 0.20 mm

#### Data collection

Bruker SMART CCD area-detector diffractometer
Absorption correction: multi-scan (*SADABS*; Bruker, 2001 ▶) *T*_min_ = 0.939, *T*_max_ = 0.947
4678 measured reflections
3267 independent reflections
2015 reflections with *I* > 2σ(*I*)
*R*_int_ = 0.032

#### Refinement

*R*[*F* ^2^ > 2σ(*F* ^2^)] = 0.061
*wR*(*F* ^2^) = 0.185
*S* = 1.02
3267 reflections
222 parameters
4 restraints
H atoms treated by a mixture of independent and constrained refinement
Δρ_max_ = 0.36 e Å^−3^
Δρ_min_ = −0.48 e Å^−3^

### 

Data collection: *SMART* (Bruker, 2007 ▶); cell refinement: *SAINT* (Bruker, 2007 ▶); data reduction: *SAINT*; program(s) used to solve structure: *SHELXTL* (Sheldrick, 2008 ▶); program(s) used to refine structure: *SHELXTL*; molecular graphics: *SHELXTL*; software used to prepare material for publication: *SHELXTL*.

## Supplementary Material

Crystal structure: contains datablock(s) global, I. DOI: 10.1107/S1600536811020769/cv5103sup1.cif

Structure factors: contains datablock(s) I. DOI: 10.1107/S1600536811020769/cv5103Isup2.hkl

Supplementary material file. DOI: 10.1107/S1600536811020769/cv5103Isup3.cml

Additional supplementary materials:  crystallographic information; 3D view; checkCIF report

## Figures and Tables

**Table 1:** Hydrogen-bond geometry (Å, °)

*D*—H⋯*A*	*D*—H	H⋯*A*	*D*⋯*A*	*D*—H⋯*A*
N2—H2⋯O5	0.90 (1)	1.92 (1)	2.813 (4)	169 (4)
O5—H5*B*⋯O1^iii^	0.84 (1)	2.03 (2)	2.783 (3)	150 (4)
O5—H5*A*⋯O1^iv^	0.84 (1)	2.30 (3)	3.004 (4)	142 (3)

Symmetry codes: (iii) 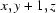; (iv) 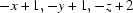.

**Fig. 1.**
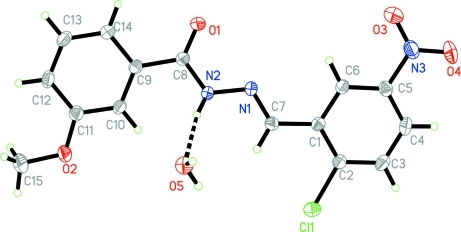
The molecular structure of (I). Displacement ellipsoids are drawn at the 30% probability level. H atoms are shown as spheres of arbitrary radius. Hydrogen bond is shown as a dashed line.

**Fig. 2.**
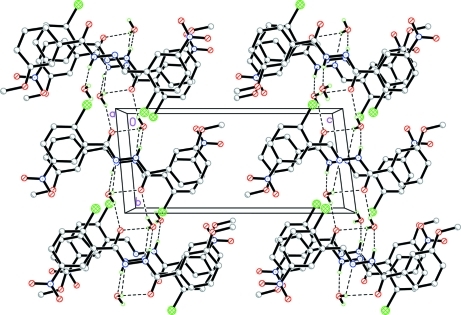
A portion of the crystal packing viewed along the a axis. Hydrogen bonds are shown as dashed lines. H atoms not involved in hydrogen bonding have been omitted.

## supplementary crystallographic information

### Comment

In continuation of our structural studies of hydrazone derivatives (Liu & You, 2010*a*,*b*,*c*; Liu & Wang, 2010*a*,*b*; Sun *et al.*, 2011), we present here the title compound (I) (Fig. 1).

In the hydrazone molecule of (I) , two benzene rings form a dihedral angle of 3.6 (1)°. In the crystal structure, the crystalline water molecules are involved in formation of intermolecular N—H···O and O—H···O hydrogen bonds (Table 2), which link the molecules into doubled ribbons extended along *b* axis (Fig. 2). Intermolecular π–π interactions (Table 1) between the aromatic rings link further these ribbons into layers parallel to *ab* plane.

### Experimental

The title compound was prepared by the condensation reaction of 2-chloro-5-nitrobenzaldehyde (1.0 mmol, 0.185 g) and 3-methoxybenzohydrazide (1.0 mmol, 0.166 g) in methanol (50 ml) at ambient temperature. Colourless block-shaped single crystals suitable for X-ray structural determination were obtained by slow evaporation of the solution for a few days.

### Refinement

N- and O-bound H atoms were located from a difference Fourier map and refined with U_iso_(H) fixed to 0.08 and with the N—H distance restrained to 0.90 (1) Å and O—H distances restrained to 0.84 (1). The remaining H atoms were positioned geometrically and constrained to ride on their parent atoms, with C—H distances of 0.93–0.96 Å, and with *U*_iso_(H) = 1.2–1.5*U*_eq_(C).

### Crystal data

**Table:**

C_15_H_12_ClN_3_O_4_·H_2_O	*Z* = 2
*M**_r_* = 351.74	*F*(000) = 364
Triclinic, *P*1	*D*_x_ = 1.504 Mg m^−^^3^
*a* = 7.176 (2) Å	Mo *K*α radiation, λ = 0.71073 Å
*b* = 7.179 (2) Å	Cell parameters from 887 reflections
*c* = 15.395 (4) Å	θ = 2.8–25.3°
α = 83.820 (18)°	µ = 0.28 mm^−^^1^
β = 89.953 (18)°	*T* = 298 K
γ = 80.190 (18)°	Block, colourless
*V* = 776.8 (4) Å^3^	0.23 × 0.21 × 0.20 mm

### Data collection

**Table:**

Bruker SMART CCD area-detector diffractometer	3267 independent reflections
Radiation source: fine-focus sealed tube	2015 reflections with *I* > 2σ(*I*)
graphite	*R*_int_ = 0.032
ω scans	θ_max_ = 27.0°, θ_min_ = 1.3°
Absorption correction: multi-scan (*SADABS*; Bruker, 2001)	*h* = −9→9
*T*_min_ = 0.939, *T*_max_ = 0.947	*k* = −8→9
4678 measured reflections	*l* = −19→19

### Refinement

**Table:**

Refinement on *F*^2^	Primary atom site location: structure-invariant direct methods
Least-squares matrix: full	Secondary atom site location: difference Fourier map
*R*[*F*^2^ > 2σ(*F*^2^)] = 0.061	Hydrogen site location: inferred from neighbouring sites
*wR*(*F*^2^) = 0.185	H atoms treated by a mixture of independent and constrained refinement
*S* = 1.02	*w* = 1/[σ^2^(*F*_o_^2^) + (0.0928*P*)^2^] where *P* = (*F*_o_^2^ + 2*F*_c_^2^)/3
3267 reflections	(Δ/σ)_max_ < 0.001
222 parameters	Δρ_max_ = 0.36 e Å^−^^3^
4 restraints	Δρ_min_ = −0.48 e Å^−^^3^

### Special details

**Table:**

Geometry. All esds (except the esd in the dihedral angle between two l.s. planes) are estimated using the full covariance matrix. The cell esds are taken into account individually in the estimation of esds in distances, angles and torsion angles; correlations between esds in cell parameters are only used when they are defined by crystal symmetry. An approximate (isotropic) treatment of cell esds is used for estimating esds involving l.s. planes.
Refinement. Refinement of F^2^ against ALL reflections. The weighted R-factor wR and goodness of fit S are based on F^2^, conventional R-factors R are based on F, with F set to zero for negative F^2^. The threshold expression of F^2^ > 2sigma(F^2^) is used only for calculating R-factors(gt) etc. and is not relevant to the choice of reflections for refinement. R-factors based on F^2^ are statistically about twice as large as those based on F, and R- factors based on ALL data will be even larger.

### Fractional atomic coordinates and isotropic or equivalent isotropic displacement parameters (Å^2^)

**Table:**

	*x*	*y*	*z*	*U*_iso_*/*U*_eq_	
Cl1	0.05788 (14)	1.03926 (12)	1.12824 (6)	0.0525 (3)	
N1	0.2400 (4)	0.5012 (4)	1.03085 (15)	0.0348 (6)	
N2	0.2787 (4)	0.5246 (3)	0.94318 (16)	0.0350 (6)	
N3	0.1926 (5)	0.3190 (5)	1.36258 (19)	0.0543 (8)	
O1	0.3018 (4)	0.2107 (3)	0.93423 (15)	0.0486 (6)	
O2	0.2513 (4)	0.8041 (4)	0.63274 (15)	0.0594 (8)	
O3	0.2584 (5)	0.1707 (4)	1.33358 (19)	0.0750 (9)	
O4	0.1633 (5)	0.3291 (4)	1.44026 (17)	0.0838 (10)	
O5	0.4570 (3)	0.8422 (3)	0.90264 (16)	0.0475 (6)*	
C1	0.1635 (4)	0.6554 (4)	1.15934 (19)	0.0322 (7)	
C2	0.0887 (4)	0.8238 (4)	1.1940 (2)	0.0350 (7)	
C3	0.0387 (5)	0.8259 (5)	1.2810 (2)	0.0435 (8)	
H3	−0.0148	0.9392	1.3021	0.052*	
C4	0.0690 (5)	0.6595 (5)	1.3356 (2)	0.0448 (8)	
H4	0.0350	0.6582	1.3940	0.054*	
C5	0.1506 (5)	0.4938 (5)	1.3029 (2)	0.0373 (8)	
C6	0.1960 (4)	0.4882 (4)	1.2159 (2)	0.0356 (7)	
H6	0.2478	0.3738	1.1954	0.043*	
C7	0.2063 (4)	0.6559 (4)	1.0662 (2)	0.0362 (7)	
H7	0.2088	0.7708	1.0323	0.043*	
C8	0.3063 (4)	0.3706 (4)	0.8984 (2)	0.0335 (7)	
C9	0.3373 (4)	0.4103 (4)	0.8024 (2)	0.0327 (7)	
C10	0.2874 (4)	0.5904 (4)	0.7584 (2)	0.0366 (7)	
H10	0.2378	0.6916	0.7892	0.044*	
C11	0.3104 (5)	0.6216 (5)	0.6691 (2)	0.0397 (8)	
C12	0.3870 (5)	0.4713 (5)	0.6231 (2)	0.0449 (8)	
H12	0.4035	0.4907	0.5631	0.054*	
C13	0.4381 (5)	0.2933 (5)	0.6678 (2)	0.0462 (9)	
H13	0.4893	0.1925	0.6371	0.055*	
C14	0.4157 (5)	0.2598 (5)	0.7561 (2)	0.0396 (8)	
H14	0.4524	0.1383	0.7849	0.048*	
C15	0.2835 (6)	0.8508 (6)	0.5424 (2)	0.0683 (12)	
H15A	0.4162	0.8194	0.5312	0.103*	
H15B	0.2414	0.9844	0.5267	0.103*	
H15C	0.2148	0.7799	0.5083	0.103*	
H2	0.334 (5)	0.624 (4)	0.923 (3)	0.080*	
H5A	0.563 (3)	0.822 (5)	0.928 (2)	0.080*	
H5B	0.382 (4)	0.931 (4)	0.921 (2)	0.080*	

### Atomic displacement parameters (Å^2^)

**Table:**

	*U*^11^	*U*^22^	*U*^33^	*U*^12^	*U*^13^	*U*^23^
Cl1	0.0666 (7)	0.0353 (5)	0.0513 (6)	0.0039 (4)	0.0017 (4)	−0.0056 (4)
N1	0.0430 (16)	0.0342 (14)	0.0266 (13)	−0.0046 (12)	0.0026 (11)	−0.0043 (11)
N2	0.0474 (17)	0.0316 (14)	0.0263 (13)	−0.0060 (12)	0.0050 (12)	−0.0053 (11)
N3	0.069 (2)	0.057 (2)	0.0384 (18)	−0.0204 (17)	−0.0057 (15)	0.0042 (15)
O1	0.0780 (18)	0.0293 (12)	0.0386 (13)	−0.0088 (12)	0.0036 (12)	−0.0046 (10)
O2	0.085 (2)	0.0501 (15)	0.0335 (13)	0.0100 (14)	0.0089 (13)	0.0051 (11)
O3	0.114 (3)	0.0452 (16)	0.0614 (19)	−0.0077 (17)	−0.0034 (17)	0.0033 (14)
O4	0.133 (3)	0.086 (2)	0.0292 (14)	−0.021 (2)	0.0034 (16)	0.0090 (14)
C1	0.0302 (16)	0.0331 (16)	0.0331 (16)	−0.0038 (13)	−0.0005 (13)	−0.0062 (13)
C2	0.0334 (17)	0.0363 (17)	0.0347 (17)	−0.0030 (13)	−0.0015 (13)	−0.0053 (13)
C3	0.044 (2)	0.045 (2)	0.0417 (19)	−0.0018 (16)	0.0014 (16)	−0.0174 (16)
C4	0.048 (2)	0.060 (2)	0.0297 (17)	−0.0132 (17)	0.0049 (15)	−0.0131 (16)
C5	0.0391 (19)	0.0415 (18)	0.0330 (17)	−0.0134 (15)	−0.0025 (14)	−0.0011 (14)
C6	0.0389 (18)	0.0356 (17)	0.0325 (16)	−0.0047 (14)	0.0004 (14)	−0.0072 (13)
C7	0.0397 (18)	0.0320 (16)	0.0342 (16)	−0.0009 (14)	0.0045 (14)	−0.0008 (13)
C8	0.0372 (18)	0.0305 (16)	0.0331 (16)	−0.0044 (13)	0.0001 (13)	−0.0059 (13)
C9	0.0309 (17)	0.0351 (16)	0.0334 (16)	−0.0064 (13)	0.0002 (13)	−0.0080 (13)
C10	0.0410 (19)	0.0355 (17)	0.0319 (16)	0.0000 (14)	0.0021 (14)	−0.0080 (13)
C11	0.0392 (19)	0.0416 (18)	0.0365 (18)	−0.0020 (15)	0.0026 (15)	−0.0036 (14)
C12	0.051 (2)	0.054 (2)	0.0320 (17)	−0.0110 (17)	0.0133 (16)	−0.0106 (15)
C13	0.056 (2)	0.0410 (19)	0.045 (2)	−0.0088 (16)	0.0150 (17)	−0.0183 (15)
C14	0.046 (2)	0.0347 (17)	0.0379 (18)	−0.0044 (15)	0.0032 (15)	−0.0066 (14)
C15	0.096 (3)	0.067 (3)	0.036 (2)	−0.005 (2)	0.004 (2)	0.0071 (19)

### Geometric parameters (Å, °)

**Table:**

Cl1—C2	1.735 (3)	C4—C5	1.380 (5)
N1—C7	1.275 (4)	C4—H4	0.9300
N1—N2	1.376 (3)	C5—C6	1.381 (4)
N2—C8	1.351 (4)	C6—H6	0.9300
N2—H2	0.902 (10)	C7—H7	0.9300
N3—O3	1.219 (4)	C8—C9	1.499 (4)
N3—O4	1.222 (4)	C9—C10	1.383 (4)
N3—C5	1.461 (4)	C9—C14	1.396 (4)
O1—C8	1.225 (3)	C10—C11	1.383 (4)
O2—C11	1.365 (4)	C10—H10	0.9300
O2—C15	1.424 (4)	C11—C12	1.389 (5)
O5—H5A	0.837 (10)	C12—C13	1.376 (5)
O5—H5B	0.835 (10)	C12—H12	0.9300
C1—C6	1.391 (4)	C13—C14	1.369 (5)
C1—C2	1.397 (4)	C13—H13	0.9300
C1—C7	1.467 (4)	C14—H14	0.9300
C2—C3	1.388 (4)	C15—H15A	0.9600
C3—C4	1.370 (5)	C15—H15B	0.9600
C3—H3	0.9300	C15—H15C	0.9600
			
Cg1···Cg2^i^	3.712 (3)	Cg1···Cg2^ii^	3.672 (3)
			
C7—N1—N2	114.2 (2)	C1—C7—H7	119.5
C8—N2—N1	119.0 (2)	O1—C8—N2	121.7 (3)
C8—N2—H2	118 (3)	O1—C8—C9	122.8 (3)
N1—N2—H2	118 (3)	N2—C8—C9	115.5 (3)
O3—N3—O4	122.8 (3)	C10—C9—C14	119.4 (3)
O3—N3—C5	119.1 (3)	C10—C9—C8	121.9 (3)
O4—N3—C5	118.1 (3)	C14—C9—C8	118.7 (3)
C11—O2—C15	118.6 (3)	C9—C10—C11	120.7 (3)
H5A—O5—H5B	113 (2)	C9—C10—H10	119.7
C6—C1—C2	117.9 (3)	C11—C10—H10	119.7
C6—C1—C7	121.3 (3)	O2—C11—C10	115.3 (3)
C2—C1—C7	120.8 (3)	O2—C11—C12	124.9 (3)
C3—C2—C1	121.9 (3)	C10—C11—C12	119.8 (3)
C3—C2—Cl1	117.9 (2)	C13—C12—C11	118.9 (3)
C1—C2—Cl1	120.2 (2)	C13—C12—H12	120.5
C4—C3—C2	119.4 (3)	C11—C12—H12	120.5
C4—C3—H3	120.3	C14—C13—C12	122.0 (3)
C2—C3—H3	120.3	C14—C13—H13	119.0
C3—C4—C5	119.1 (3)	C12—C13—H13	119.0
C3—C4—H4	120.5	C13—C14—C9	119.2 (3)
C5—C4—H4	120.5	C13—C14—H14	120.4
C4—C5—C6	122.3 (3)	C9—C14—H14	120.4
C4—C5—N3	118.8 (3)	O2—C15—H15A	109.5
C6—C5—N3	119.0 (3)	O2—C15—H15B	109.5
C5—C6—C1	119.3 (3)	H15A—C15—H15B	109.5
C5—C6—H6	120.3	O2—C15—H15C	109.5
C1—C6—H6	120.3	H15A—C15—H15C	109.5
N1—C7—C1	121.0 (3)	H15B—C15—H15C	109.5
N1—C7—H7	119.5		

Symmetry codes: (i) −*x*+1, −*y*+1, −*z*; (ii) −*x*+2, −*y*+1, −*z*.

### Hydrogen-bond geometry (Å, °)

**Table:**

*D*—H···*A*	*D*—H	H···*A*	*D*···*A*	*D*—H···*A*
N2—H2···O5	0.90 (1)	1.92 (1)	2.813 (4)	169 (4)
O5—H5B···O1^iii^	0.84 (1)	2.03 (2)	2.783 (3)	150 (4)
O5—H5A···O1^iv^	0.84 (1)	2.30 (3)	3.004 (4)	142 (3)

Symmetry codes: (iii) *x*, *y*+1, *z*; (iv) −*x*+1, −*y*+1, −*z*+2.
